# Trends in the usage of adjuvant systemic therapy for breast cancer in the Netherlands and its effect on mortality

**DOI:** 10.1038/sj.bjc.6601969

**Published:** 2004-06-22

**Authors:** M M Vervoort, G Draisma, J Fracheboud, L V van de Poll-Franse, H J de Koning

**Affiliations:** 1 Department of Public Health, Erasmus MC, University Medical Center Rotterdam, PO Box 1738, 3000 DR Rotterdam, The Netherlands; 2 Netherlands Institute for Health Sciences, PO Box 1738, 3000 DR Rotterdam, The Netherlands; 3 Comprehensive Cancer Centre South (IKZ), PO Box 231, 5600 AE Eindhoven, The Netherlands

**Keywords:** breast cancer, adjuvant therapy, mortality, mammography screening

## Abstract

Adjuvant systemic therapy was introduced in the Netherlands as a breast cancer treatment in the early 1980s. In this paper, we describe the trends in the usage of adjuvant systemic treatment in the period 1975–1997 in the Netherlands. The main aim of our study was to assess the effects of adjuvant tamoxifen and polychemotherapy on breast cancer mortality, compared to the effects of the mammography screening programme. The computer simulation model MIcrosimulation SCreening ANalysis, which simulates demography, natural history of breast cancer and screening effects, was used to estimate the effects. Use of adjuvant therapy increased over time, but since 1990 it remained rather stable. Nowadays, adjuvant therapy is given to 88% of node-positive patients aged 50–69 years, while less than 10% of node-negative patients receive any kind of adjuvant treatment. Adjuvant treatment is given independent of the mode of detection (adjusted by nodal status and size). We predict that the reduction in breast cancer mortality due to adjuvant therapy is 7% in women aged 55–74 years, while the reduction due to screening, which was first implemented in women aged 50–69 years in 1990–97, will be 28–30% in 2007. In conclusion, although adjuvant systemic therapy can reduce breast cancer mortality rates, it is anticipated to be less than the mortality reduction caused by mammography screening.

The use of adjuvant polychemotherapy and hormonal therapy for early breast cancer has strongly increased over the last two decades. This often happened subsequent to new data from the worldwide overview analyses of randomised clinical trials assessing the effects of adjuvant therapy, showing significant benefits in disease-free and overall survival (EBCTCG, 1992, 1998a, 1998b).

In 2000, the Dutch National Breast Cancer Platform (NABON) and the Dutch Society for Medical Oncology (NVMO) developed a consensus guideline for adjuvant systemic therapy (Bontenbal *et al*, 2000). It recommended tamoxifen use for patients with node-positive tumours and a positive oestrogen and/or progesterone receptor status. Chemotherapy was recommended for all node-positive tumours in premenopausal women and in postmenopausal women under age 70, with a negative hormonal receptor status. For node-negative tumours, the recommendation of adjuvant therapy depends on the tumour size, differentiation grade and mitotic activity index. Before the introduction of this new guideline, tamoxifen was mainly recommended for postmenopausal women with node-positive tumours and chemotherapy for premenopausal women with node-positive tumours.

Only few data are available on the use of adjuvant therapy in different countries, including the Netherlands, over the last two decades. More information about its use and effects would be needed in order to explain what part of the recent mortality reduction in breast cancer patients in the Netherlands may be caused by changes in adjuvant therapy and what part can be contributed to the Dutch breast cancer screening programme (Otto *et al*, 2003).

The main objective of this study is to assess the potential effects of adjuvant polychemotherapy and adjuvant tamoxifen on breast cancer mortality in the Netherlands using the microsimulation model MIcrosimulation SCreening ANalysis (MISCAN) (De Koning *et al*, 1995). This model simulates the future demographic characteristics of the Dutch population, the natural history of breast cancer and screening effects. We assess in this paper the trends in the usage of adjuvant polychemotherapy and adjuvant tamoxifen for the treatment of breast cancer in the Netherlands from 1975 until 1997, and estimate its potential impact on mortality.

## MATERIALS AND METHODS

### Data sources

The Dutch national biannual breast cancer screening programme started around 1990 and covered in 1997 all women aged 50–69 years. Data from all nine regions, carrying out the screening programme, have been collected and analysed since 1990 by the National Evaluation Team for Breast cancer screening (NETB), that annually reports in the Netherlands on population, screen invitations and examinations, breast cancers diagnosed, interval cancers and breast cancer therapy (Fracheboud *et al*, 2001). Detailed data on the use of adjuvant systemic therapy were obtained from the NETB and the Eindhoven Cancer Registry (ECR).

The Southeast Netherlands, with a population of almost one million inhabitants (6% of the Dutch population), is covered by the population-based regional Eindhoven Cancer Registry, which collected data from pathology reports and clinical records on all cancer patients since 1955 according to international guidelines (Voogd *et al*, 1994). It is part of the Dutch Cancer Registry since 1989. We used a data set on adjuvant therapy from 1975 until 1997. Patients were staged according to the TNM system.

### Description of the MISCAN model

The computer simulation package MISCAN was used to assess the effects of adjuvant systemic therapy on breast cancer mortality. A full description of the MISCAN model has been published before (van Oortmarssen *et al*, 1990; De Koning *et al*, 1995). In brief, the model first simulates individual life histories for women in the absence of screening and then assesses how these histories would change as a consequence of a screening program. The natural history is modelled as a progression from no breast cancer through preclinical disease to clinical disease.

The different disease states are: no breast cancer, five preclinical disease states, namely Ductal Carcinoma In Situ (DCIS) and four invasive preclinical states according to tumour size (<0.5, 0.6–1.0, 1.1–2.0 and >2.0 cm). From a given preclinical state, a cancer may be detected by screening, or become clinically apparent or, if undiagnosed, progress to the next preclinical state. Parameters of the preclinical phase such as transition probabilities between states, mean durations and sensitivities of the screening test were based on data from the Dutch screening programme and the pilot studies in Utrecht and Nijmegen (van Oortmarssen *et al*, 1990; LETB, 1994). After a diagnosis of breast cancer, the survival period depends on the disease state and age at time of diagnosis. Survival parameters have been estimated from the pilot studies. In the breast cancer model, women with screen-detected cancers can have a reduced risk of dying of breast cancer depending on the detected cancer size. Stage-specific cure rates of screen-detected cancers have been estimated from the published data of the Swedish trials (Nyström *et al*, 1993; De Koning *et al*, 1995). The model output includes the number of screen-detected and clinically detected cancers and their stage distributions, the clinical age-specific breast cancer incidence by stage and the age-specific breast cancer mortality, all for both the situation with and without screening.

The programme has been used extensively to analyse screening in various settings (van Ineveld *et al*, 1993; Paci *et al*, 1995; van den Akker-van Marle *et al*, 1997) and to predict mortality trends (van den Akker-van Marle *et al*, 1999).

### Adaptations in model

For the present purpose, we extended the breast cancer model with lymph node status: during tumour growth, breast cancer can change from node negative (N−) to node positive (N+). Transition probabilities were estimated from data of the national screening programme (LETB, 1994). Tumour size and lymph node status-specific survival were estimated from the Utrecht pilot study of screening (Collette *et al*, 1992). This pilot study was conducted mainly before the introduction of adjuvant therapy, and therefore is a good starting point to estimate the contribution of adjuvant therapy to observed mortality trends. The extended model reproduced the incidence, stage distribution and mortality from breast cancer in the Netherlands satisfactorily.

The benefit of adjuvant therapy in reducing breast cancer mortality was included in the model using the published proportional reductions in annual odds of death from adjuvant tamoxifen and polychemotherapy from the 1998 Early Breast Cancer Trialists' Collaborative Group (EBCTCG) meta-analysis of clinical trials (EBCTCG, 1998a, 1998b). We used these hazard ratios to predict the effects of tamoxifen and polychemotherapy on breast cancer mortality (Table 1

**Table 1:** Hazard ratios and mean proportions of women using adjuvant therapy in 1990–97 in different MISCAN models

	**Hazard ratio**		**Proportion of women using adjuvant therapy (proportion of total number of women with breast cancer)**	
	**N−**	**N+**	**N−**	**N+**
Treatment and age				
Tamoxifen for 2 years, age <50	0.89^a^	0.81^a^	0.0^b^ (16.0)	0.1^b^ (13.0)
Tamoxifen for 2 years, age 50–69	0.89^a^	0.81^a^	0.065^b^ (32.0)	0.78^b^ (19.0)
Tamoxifen for 2 years, age 70+	0.89^a^	0.81^a^	0.065^b^ (12.0)	0.85^b^ (8.0)
Chemotherapy, age <50	0.73^a^	0.73^a^	0.02^b^ (16.0)	0.70^b^ (13.0)
Chemotherapy, age 50–69	0.89^*^	0.89^a^	0.01^b^ (32.0)	0.12^b^ (19.0)
Chemotherapy, age 70+	1.0	1.0	0.0^b^ (12.0)	0.01^b^ (8.0)

a Data were obtained from the 1998 Early Breast Cancer Trialists' Collaborative Group meta-analysis of clinical trials (EBCTCG, 1998a, b).

b Data were obtained from the NETB and the Eindhoven Cancer Registry.

).

### Analysis model

We established three different models, the first one without the effects of adjuvant therapy (which consists of the MISCAN model including nodal status), the second including effects of chemotherapy and the third model including the effects of tamoxifen. We predicted breast cancer mortality rates per 100 000 person-years, using the number of deaths from breast cancer as the numerator and the female population as the denominator. We compared the mortality rates for three different age groups, 45–54, 55–64 and 65–74 years. Here, age is specified as age at death. MISCAN predicts mortality rates for the period 1986–2015 for situations with and without adjuvant therapy (chemotherapy and tamoxifen together), and in which a screening programme does and does not exist.

In order to calculate the mortality rates for women using adjuvant therapy, we took weighted averages of the number of breast cancer deaths and life-years in each of the three models. Subsequently, we divided the number of deaths by life-years lived to calculate the mortality rates. The weights are the mean proportions of women by age, who received tamoxifen or polychemotherapy or none in 1990–97 (Table 1). Although adjuvant therapy was already introduced at a small scale in the early 1980s, we used data of the period 1990–97, because the use of adjuvant therapy remained rather stable since 1990 and we assumed a constant use of adjuvant therapy.

## RESULTS

Figure 1A–C

**Figure 1:**
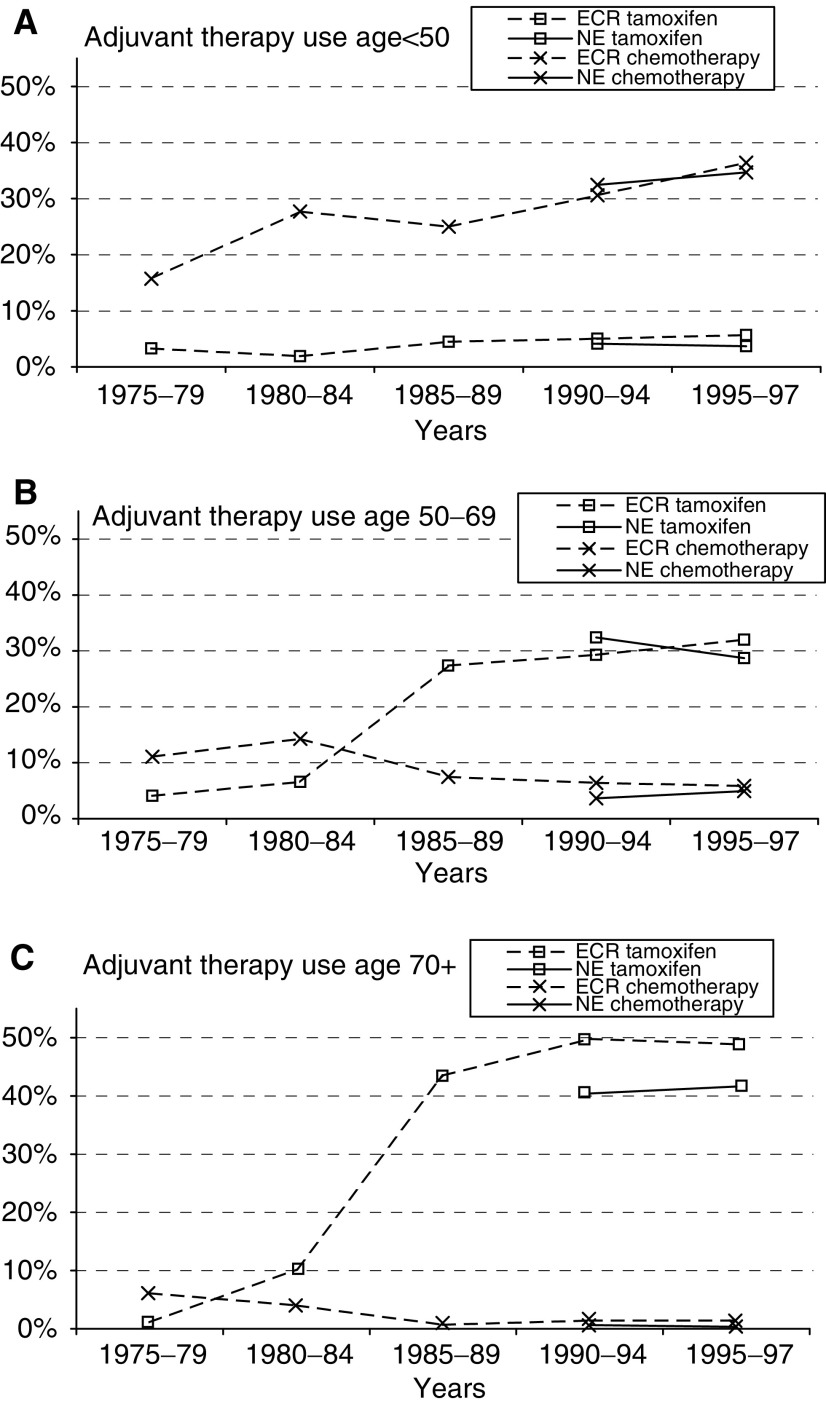
(**A–C**) Trends in the usage of tamoxifen and chemotherapy in 1975–97 in the Southeast region (ECR) and all regions of the Netherlands (NE), subdivided by age (*source*: Eindhoven Cancer Registry and NETB).

illustrates the trends in the use of hormonal therapy and chemotherapy for women diagnosed with breast cancer at age <50, 50–69 and 70+ in the years 1975–97. In the age group 50–69, the data from the Southeast Netherlands and the Netherlands are comparable in the overlapping period 1990–97. In the age group >70, the Eindhoven Cancer Registry reported a somewhat higher use of both tamoxifen and chemotherapy than the Netherlands as a whole in the period 1990–97. The proportion of patients receiving adjuvant chemotherapy increased in 1980–84, especially in the age group <50, but also in the age group 50–69, and thereafter decreased in 1985–89. In the latter period, there was a large increase in the treatment with tamoxifen for all age groups. In 1990–97, the use of adjuvant tamoxifen and chemotherapy remained rather stable in the Netherlands.

Table 2

**Table 2:** Adjuvant therapy by screening status and nodal status for age group 50–69 years in 1990–1997

**Period**	**N− (N)**	**H (%)**	**C (%)**	**H+C (%)**	**All (%)**	**N+ (N)**	**H (%)**	**C (%)**	**H+C (%)**	**All (%)**	**All tumours (N)**	**H (%)**	**C (%)**	**H+C (%)**	**All (%)**
*Screen-detected*															
1990	180	2.2	0.0	0.0	2.2	64	75.0	3.1	0.0	78.1	244	21.3	0.8	0.0	22.1
1991	422	6.6	0.2	0.0	6.8	170	62.9	10.0	7.1	80.0	592	22.8	3.0	2.0	27.9
1992	798	8.3	0.0	0.0	8.3	307	73.9	6.8	2.9	83.6	1105	26.5	1.9	0.8	29.2
1993	985	7.4	0.1	0.0	7.5	343	74.9	4.4	4.4	83.7	1328	24.8	1.2	1.1	27.2
1994	1160	2.8	0.1	0.0	2.9	376	77.4	5.3	2.4	85.1	1536	21.1	1.4	0.6	23.0
1995	1146	3.6	0.4	0.1	4.1	415	79.3	7.2	2.9	89.4	1561	23.7	2.2	0.8	26.8
1996	1293	3.8	0.3	0.0	4.1	442	75.6	9.7	3.9	89.2	1735	22.1	2.7	1.0	25.8
1997	1300	4.9	0.9	0.1	5.9	448	78.4	16.1	3.6	98.1	1748	23.7	4.7	1.0	29.4
1990–97	7284	4.9	0.3	0.0	5.2	2565	75.8	8.6	3.5	87.9	9849	23.4	2.5	0.9	26.8
1990–97	T1N0	4.6	0.2	0.0	4.8	T1N1	74.9	8.7	3.3	86.9					
	T2+N0	9.3	1.2	0.2	10.7	T2+N1	76.3	8.3	3.7	88.3					
															
*Not screen-detected*															
1990	1062	8.2	0.9	0.7	9.8	792	72.7	5.6	1.9	80.2	1854	35.8	2.9	1.2	39.8
1991	1171	11.2	1.0	0.6	12.8	853	76.9	11.3	1.3	89.5	2024	38.9	5.3	0.9	45.1
1992	1227	10.3	1.6	1.0	12.9	901	75.0	7.1	2.8	84.9	2128	37.7	3.9	1.7	43.4
1993	1194	9.1	1.8	0.9	11.8	850	73.9	9.8	4.0	87.7	2044	36.1	5.1	2.2	43.3
1994	1343	5.1	1.9	1.4	8.4	1027	72.4	12.1	3.9	88.4	2370	34.3	6.3	2.5	43.0
1995	1088	4.7	0.8	0.3	5.8	836	76.0	11.1	3.2	90.3	1924	35.7	5.3	1.6	42.5
1996	1040	6.1	0.7	0.0	6.8	757	71.5	13.2	4.8	89.5	1797	33.6	6.0	2.0	41.6
1997	1042	7.4	1.7	0.6	9.7	758	70.6	17.7	6.3	94.6	1800	34.0	8.4	3.0	45.4
1990–97	9167	7.8	1.3	0.7	9.8	6774	73.7	10.9	3.5	88.1	15 941	35.8	5.4	1.9	43.1
1990–97	T1N0	6.9	1.1	0.8	8.8	T1N1	74.9	10.4	2.9	88.2					
	T2+N0	12.2	2.2	0.8	15.2	T2+N1	73.5	11.6	3.9	89.0					

*Source*: NETB. H=hormonal therapy, C=chemotherapy.

summarises the use of adjuvant therapy by detection status and nodal status for women aged 50–69 in 1990–97. Adjuvant chemotherapy was mainly administered to node-positive premenopausal patients and adjuvant tamoxifen to node-positive postmenopausal patients in 1975–97. The use of adjuvant systemic therapy did not depend on screen status. In both screened and not-screened women with node-positive tumours, 88% received adjuvant therapy. In 1990–97, 5.2% of the patients with node-negative screen-detected cancers and 9.8% with node-negative clinically detected cancers received adjuvant therapy (*P*=0.219). In node-positive patients, adjuvant therapy did not depend on tumour size, whereas in node-negative patients use of adjuvant tamoxifen increased as tumour size increased.

Figure 2A–C

**Figure 2:**
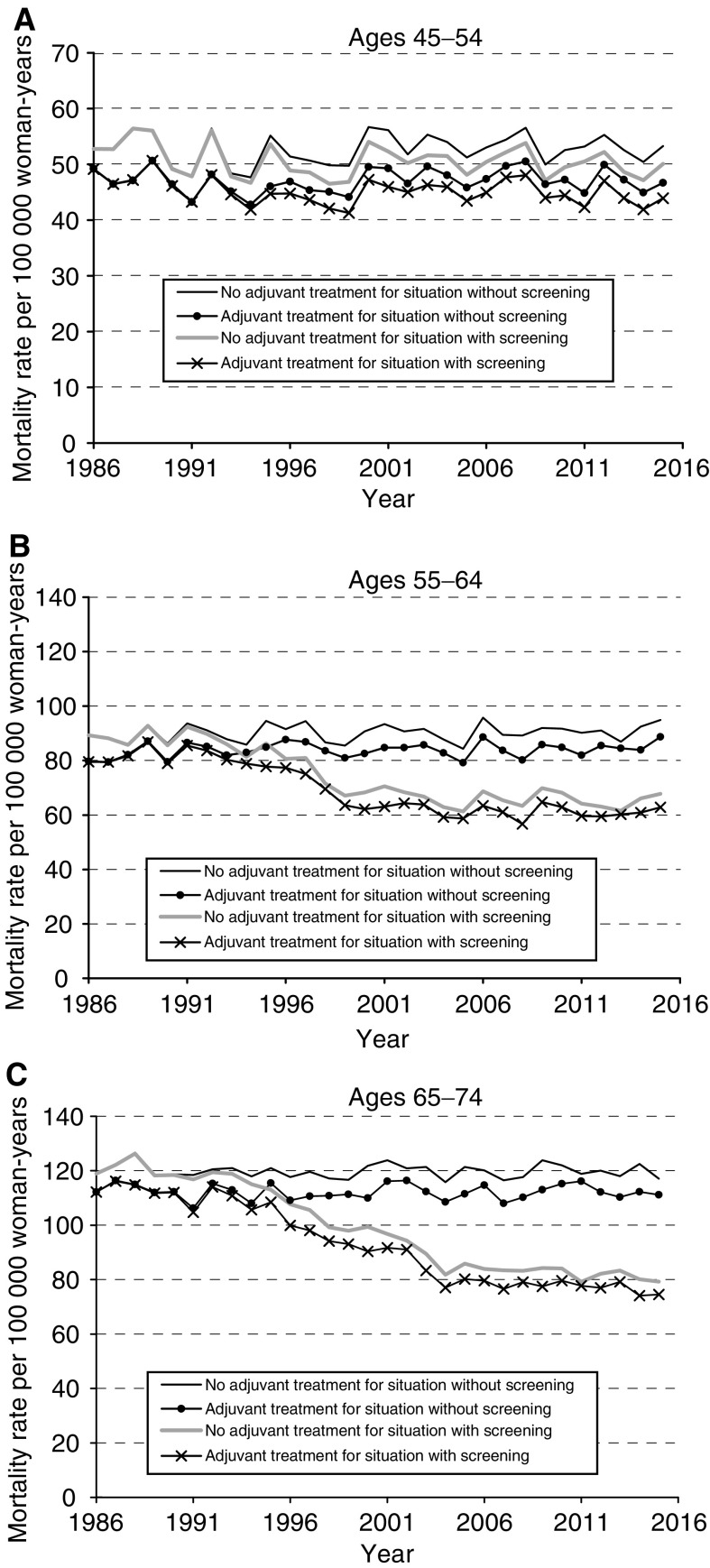
Breast cancer mortality rates predicted by MISCAN for effects of screening and adjuvant therapy (chemotherapy and tamoxifen). (**A**) Age group 45–54 (assumed first screening attendance rate of 79% in the age group 50–54). (**B**) Age group 55–64 (assumed first screening attendance rate of 76–79% in the age group 55–64). (**C**) Age group 65–74 (assumed first screening attendance rate of 72–74% in the age group 65–69).

illustrates breast cancer mortality rates predicted by MISCAN, both for the situation with and without screening and adjuvant therapy. In women aged 55–74, the mortality reduction due to adjuvant therapy is 7% in the situation without screening and 6% in the situation with screening. At 10 years after the screening programme is fully implemented (2007), and the maximum screening effect is reached, the predicted breast cancer mortality reduction due to screening would be 28–30%. Of the estimated total breast cancer mortality reduction of 34% in women aged 55–74, approximately 80% would then be explained by screening, whereas 20% would contribute to the use of adjuvant therapy. The predicted mortality rates in unscreened women aged 45–54 in the situation with and without adjuvant therapy use are 47 and 53 per 100 000 person-years, respectively. Thus, the mortality reduction due to adjuvant therapy would be 11%. The mortality reduction due to screening, which was first implemented in women aged 50–69, would be 5%. Thus, in the age group 45–54, less than 30% of the total breast cancer mortality reduction could be explained by screening, whereas 70% could be attributed to adjuvant therapy use. We assumed tamoxifen treatment for 2 years, because this was the common treatment in the Netherlands. We also predicted mortality rates for tamoxifen treatment for 5 years (data not shown), and found that the breast cancer mortality reduction due to adjuvant therapy would then be 10% in the age group 55–74.

## DISCUSSION

Our study suggests that the breast cancer mortality reduction caused by present-day practice of adjuvant tamoxifen and chemotherapy is 7%. This effect is about four times smaller than the reduction caused by breast cancer screening, estimated to amount 28–30% in 2007. This indicates that the screening programme in the Netherlands has contributed most to the recent reduction in breast cancer mortality in women aged 50–69, although adjuvant therapy could also have played an important role, particularly in the early 1990s. As most of the women who died at age 45–54 have not participated in the screening programme, the mortality reduction due to adjuvant treatment in this age group must be larger than due to screening. Tamoxifen contributes most to the mortality reduction from adjuvant therapy in women aged 55–74, because it is prescribed to mainly postmenopausal women. In this age group, less than 10% of node-positive women and less than 1% of node-negative women receive chemotherapy, so the overall effect of chemotherapy on mortality is very small. The effect of chemotherapy is particularly seen in the age group <50.

Adjuvant systemic therapy was introduced and used on a large scale before the implementation of the screening programme. There has been little change in the use of adjuvant therapy since 1990 and the maximum mortality reduction by adjuvant therapy was expected to appear already in the first years after its maximum use (in 1990–94). In the case of screening, a significant mortality reduction is expected some years after 1997 (Otto *et al*, 2003), the year in which the screening programme was fully implemented. Therefore, the mortality-lowering effect of the increased use of adjuvant treatment in the 1980s is expected to occur before the effect of the screening programme.

Since we adjusted survival after clinical diagnosis in MISCAN, the use and effects of adjuvant therapy are the same for clinically detected cancers and screen-detected cancers, despite earlier detection and improvement in prognosis of the latter. As screening leads to the detection of smaller tumour sizes and these tumours are less likely to receive adjuvant therapy, it can be argued that this will lead to a decline in the use of adjuvant therapy in screen-detected cancers.

In a previous study in England and Wales, Blanks *et al* (2000) estimated that screening contributed one-third, and other factors (including improved treatment with adjuvant systemic therapy) two-thirds to the total breast cancer mortality reduction. They argued that the estimated mortality reduction from screening may be lower than the mortality reduction predicted by MISCAN (van den Akker-van Marle *et al*, 1999) because of the lower sensitivity of the UK screening programme in the early years of screening. It has been suggested that, in the UK, there were substantial changes in adjuvant therapy and other breast cancer treatment in the mid-1980s, compared to the Netherlands. Moreover, it is likely that primary treatment as such changed considerably in the UK, whereas in the Netherlands primary treatment including radiation treatment was already practised according to guidelines (Bontenbal *et al*, 2000).

Approximately 70% of all breast cancer deaths are in patients with involved axillary nodes at diagnosis. Using this percentage and the proportions of women receiving tamoxifen or polychemotherapy and the odds reductions in mortality from the EBCTCG trial (Table 1), we calculated that the expected mortality reduction due to adjuvant treatment in women aged 50–69 should be approximately (0.7 × 0.78 × 0.19)+(0.3 × 0.065 × 0.11)+(0.7 × 0.12 × 0.11)+(0.3 × 0.01 × 0.11)=11.5%. However, MISCAN predicted that the mean mortality reduction from adjuvant therapy was 7.3% in this age group. For the age group 45–49, we calculated a mortality reduction from adjuvant therapy of 14.6%, whereas MISCAN predicted a mortality reduction of 12.4%. The explanation for this lower than expected mortality reduction, predicted by MISCAN, is as follows. The effect of adjuvant therapy on survival after clinical diagnosis was modelled assuming a constant ratio between the hazards (at time *t* since diagnosis) of dying of breast cancer in the treated and untreated groups. A hazard ratio (*r*) of 0.8, however, does not translate in a mortality ratio of 0.8. A numerical example: take *r*=0.8 and take a 10-years survival without adjuvant therapy of 0.4. Then, the 10-year survival with adjuvant therapy equals (0.4)^0.8=0.48 and the mortality with adjuvant therapy 0.52 and without therapy 0.6. The mortality ratio can be calculated as 0.52/0.6=0.87. A hazard ratio of 0.8 thus leads to a mortality ratio of 0.87, so the mortality reduction will be 13% (in stead of 20%). The smaller the tumour size and thus the better the survival, the smaller the difference between the hazard ratio and the mortality ratio.

Several factors may have led to an underestimation of the effect of adjuvant systemic therapy. Firstly, the recent introduction of new guidelines in 2000 for treatment of node-negative cancers will lead to an increase in the use of adjuvant therapy in the next few years. Therefore, our predicted mortality reductions could be an underestimation of the actual effect of adjuvant therapy in the future. Secondly, since 1990 there is a shift in the Netherlands from short-term (1–2 years) to long-term use (5 years) of tamoxifen. Furthermore, the application of adjuvant therapy has improved. Therefore, it could be argued that the expected mortality reduction from adjuvant therapy will be somewhat higher than we predicted.

There was a four-fold increase in the use of tamoxifen in the period 1985–89. This increase can be explained by the publication in 1988 of an overview of several trials on tamoxifen, which showed a significant mortality reduction if tamoxifen was used in postmenopausal node-positive patients (EBCTCG, 1988). Moreover, this drug was prescribed increasingly to women with a negative hormonal receptor status. A study in the Southeast Netherlands showed an increase in the use of tamoxifen for node-positive postmenopausal patients with oestrogen receptor-negative tumours from 0% in 1986–87 to 53% in 1990–91 (Voogd *et al*, 1994). In the case of oestrogen receptor-positive tumours, tamoxifen use also increased. Several trials have investigated the relation between hormonal receptor status and the effect of tamoxifen, but different results have been published. Although the 1985 NIH consensus conference concluded that tamoxifen should be given only to women with ER-positive tumours, the 1992 EBCTCG overview showed significant mortality reductions for women older than 50, even for those with tumours classified as oestrogen poor (EBCTCG, 1992). The conclusion from the EBCTCG overview in 1998 was that all postmenopausal women with a positive hormonal receptor status should receive hormonal therapy and that there was no clear benefit from tamoxifen in women with oestrogen and progesterone receptor-negative tumours (EBCTCG, 1998b). This has influenced the new guidelines, which do not recommend tamoxifen for hormonal receptor-negative tumours.

In conclusion, the use of adjuvant therapy strongly increased in the period 1975–97. It contributed considerably to the breast cancer mortality reduction, observed in several countries. Nevertheless, based on estimated calculations, it is good to realise that screening has an (additional) effect that in the Netherlands may be three to four times as large.

## Appendix A

Data regarding the trends in usage of adjuvant therapy are shown in Table A1

**Table A1:** Trends in the usage of adjuvant therapy by age and nodal status in the Netherlands

		**Breast cancers (*N*)**	**Breast cancers (*N*)**	**C (%)**	**H (%)**	**Breast cancers (*N*)**	**C (%)**	**H (%)**	**Breast cancers (*N*)**	**C (%)**	**H (%)**
**Period**	**Stage**	**All ages**	**Age<50**	**Age <50**	**Age <50**	**Age 50–69**	**Age 50–69**	**Age 50–69**	**Age 70+**	**Age 70+**	**Age 70+**
*ECR*											
1975–80	N−	657	219	5.5	0.0	305	4.3	2.0	133	1.5	0.0
1980–84	N−	886	292	1.7	0.0	380	2.1	0.3	214	0.5	2.3
1985–89	N−	1479	478	1.7	0.2	690	0.6	3.0	311	0.0	7.4
1990–94	N−	2829	748	2.3	0.4	1,522	0.3	3.0	559	0.0	7.2
1995–97	N−	1898	486	1.9	0.4	1,027	0.5	1.3	385	0.0	5.7
1975–80	N+	617	192	26.6	6.8	318	14.8	7.6	107	4.7	2.8
1980–84	N+	741	236	58.1	2.5	353	22.4	12.5	152	5.3	9.9
1985–89	N+	1165	381	53.0	7.6	582	11.9	51.2	202	0.5	59.4
1990–94	N+	1972	646	60.2	7.7	919	10.8	71.0	407	1.7	80.8
1995–97	N+	1220	386	77.7	11.7	562	12.3	77.9	272	0.7	92.7
1975–80	All	1631	492	15.7	3.3	777	11.1	4.1	362	6.1	1.1
1980–84	All	1890	574	27.7	1.9	818	14.3	6.6	498	4.0	10.2
1985–89	All	3157	916	25.0	4.5	1430	7.5	27.4	811	0.7	43.5
1990–94	All	5580	1488	30.6	5.0	2665	6.4	29.3	1427	1.4	49.8
1995–97	All	3608	927	36.4	5.7	1751	5.9	32.0	930	1.4	48.9
											
*NE*											
1990	All	6093	1692	28.5	3.9	2733	3.4	32.7	1668	1.1	38.3
1991	All	6520	1828	34.0	3.7	3085	4.7	34.9	1607	1.3	40.3
1992	All	7021	1886	32.9	3.8	3385	3.1	34.5	1750	0.2	42.1
1993	All	7325	1962	33.5	4.6	3583	3.1	31.5	1780	0.4	41.9
1994	All	8973	2436	32.9	4.5	4136	3.9	29.5	2365	0.3	39.6
1995	All	7844	2169	34.1	3.9	3669	4.0	30.4	2006	0.2	39.8
1996	All	8190	2347	34.1	3.5	3720	4.3	28.3	2123	0.3	42.8
1997	All	8275	2316	35.8	3.7	3880	6.4	27.5	2079	0.4	42.1
1990–94	All	35 932	9804	32.5	4.1	16 922	3.7	32.4	9170	0.6	40.4
1995–97	All	24 309	6832	34.7	3.7	11 269	4.9	28.7	6208	0.3	41.6

*Source*: NETB and Eindhoven Cancer Registry. C=chemotherapy, H=hormonal therapy.

.
